# An enzymatic tandem reaction to produce odor-active fatty aldehydes

**DOI:** 10.1007/s00253-022-12134-3

**Published:** 2022-08-30

**Authors:** Jean-Philippe Kanter, Philipp Jakob Honold, David Lüke, Sven Heiles, Bernhard Spengler, Marco Alexander Fraatz, Christoph Harms, Jakob Peter Ley, Holger Zorn, Andreas Klaus Hammer

**Affiliations:** 1 Institute of Food Chemistry and Food Biotechnology, Justus Liebig University Giessen, Heinrich-Buff-Ring 17, 35392 Giessen, Germany; 2 Institute of Inorganic and Analytical Chemistry, Justus Liebig University Giessen, Heinrich-Buff-Ring 17, 35392 Giessen, Germany; 3 Fraunhofer Institute for Molecular Biology and Applied Ecology, Ohlebergsweg 12, 35394 Giessen, Germany; 4 Symrise AG, Muehlenfeldstrasse 1, 37603 Holzminden, Germany

**Keywords:** *α*-Dioxygenase (*α*-DOX), Fatty aldehyde dehydrogenase (FALDH), Biotransformation, Fatty aldehydes, Flavoring production

## Abstract

**Abstract:**

Aldehydes represent a versatile and favored class of flavoring substances. A biocatalytic access to odor-active aldehydes was developed by conversion of fatty acids with two enzymes of the *α*-dioxygenase pathway. The recombinant enzymes *α*-dioxygenase (*α*-DOX) originating from *Crocosphaera subtropica* and fatty aldehyde dehydrogenase (FALDH) from *Vibrio harveyi* were heterologously expressed in *E. coli*, purified, and applied in a coupled (tandem) repetitive reaction. The concept was optimized in terms of number of reaction cycles and production yields. Up to five cycles and aldehyde yields of up to 26% were achieved. Afterward, the approach was applied to sea buckthorn pulp oil as raw material for the enzyme catalyzed production of flavoring/fragrance ingredients based on complex aldehyde mixtures. The most abundant fatty acids in sea buckthorn pulp oil, namely palmitic, palmitoleic, oleic, and linoleic acid, were used as substrates for further biotransformation experiments. Various aldehydes were identified, semi-quantified, and sensorially characterized by means of headspace–solid phase microextraction–gas chromatography–mass spectrometry–olfactometry (HS–SPME–GC–MS–O). Structural validation of unsaturated aldehydes in terms of double-bond positions was performed by multidimensional high-resolution mass spectrometry experiments of their Paternò–Büchi (PB) photoproducts. Retention indices and odor impressions of *inter alia* (*Z,Z*)-5,8-tetradecadienal (*Z,Z*)-6,9-pentadecadienal, (*Z*)-8-pentadecenal, (*Z*)-4-tridecenal, (*Z*)-6-pentadecenal, and (*Z*)-8-heptadecenal were determined for the first time.

**Key points:**

• *Coupled reaction of Csα-DOX and VhFALDH yields chain-shortened fatty aldehydes.*

• *Odors of several Z-unsaturated fatty aldehydes are described for the first time.*

• *Potential for industrial production of aldehyde-based odorants from natural sources.*

**Graphical abstract:**

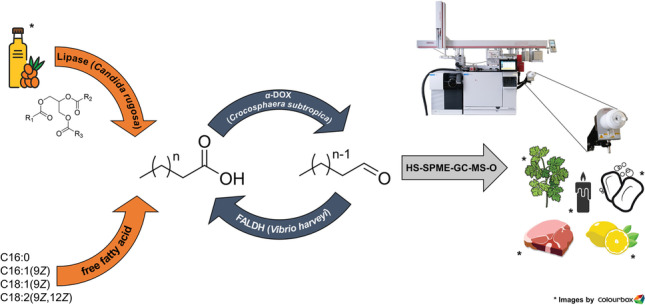

**Supplementary Information:**

The online version contains supplementary material available at 10.1007/s00253-022-12134-3.

## Introduction

Medium- and long-chain fatty aldehydes represent an important class of substances, widely applied for flavor and fragrance applications (Truong et al. 2017; Kim et al. 2022a). Saturated and unsaturated fatty aldehydes with carbon chain lengths between 11 and 18 exhibit floral, soapy, citrus-like, and waxy odors (Guadagni et al. 1963; Buttery et al. 1988). Even though fatty aldehydes are found in a wide variety of organisms, their concentrations are typically rather low and extraction from, *e.g.,* citrus peel lacks economic efficiency. Besides, due to their similar physicochemical properties, fractionation and separation of saturated and unsaturated fatty aldehydes requires costly and process-intensive techniques. On the other hand, chemical synthesis, as a rather traditional and convenient industrial method, increasingly fades from the spotlight due to several aspects. One of the main drawbacks is the trend toward natural ingredients caused by the rising consumer awareness for sustainability, ecology, and health issues. Apart from considerable skepticism for chemically synthesized food and cosmetic ingredients, such production methods still typically require augmented amounts of chemicals, often originating from petroleum and its derivatives (Burger et al. 2019).

In order to find alternative ways for the synthesis of fatty aldehydes, several approaches in the field of biotechnology have aroused. However, they mostly showed limited yields or restricted substrate specificity (Buchhaupt et al. 2012; Kerler et al. 2005). Among them, multiple enzymatic systems were applied. Lipoxygenase (LOX), which is commonly present in plant tissue, catalyzes the conversion of fatty acids to short- to medium-chained aldehydes. Kerler et al. (2005) applied a soy-derived LOX for biotransformation of hydrolyzed triglycerides or free fatty acids to short-chain aldehydes via hydroperoxides and thermal treatment under acidic conditions. Zhu et al. (2018) applied a multifunctional LOX from the algae *Pyropia haitanensis* expressed in *E. coli* for the production of C_5_–C_9 _aldehydes. Buchhaupt et al. (2012) obtained ~ 60 mg/L of C_6_-aldehydes from biotransformation of fatty acids via co-expression of a recombinant LOX and hydroperoxide lyase in *Saccharomyces cerevisiae*. Another approach makes use of direct reduction of fatty acids to the corresponding aldehydes by means of carboxylic acid reductase (CAR) (Fraatz et al. 2018; Horvat and Winkler 2020; Hammer et al. 2021). The opposite reaction from alcohols to aldehydes catalyzed by alcohol dehydrogenases has been established as well (Berger 1995). Alcohol dehydrogenases are dependent on the cofactor NAD^+^ and CARs require NADPH and additionally ATP. Therefore, cofactor regeneration is essential for large-scale biotechnological applications. In contrast, *α*-dioxygenase solely requires molecular oxygen for the catalytic *α*-oxidation of fatty acids. The resulting 2-hydroperoxy fatty acid either reacts to a 2-hydroxy fatty acid (C_n_) or spontaneously decarboxylates, forming a C_n-1_ aldehyde (Hamberg et al. 2002; Kim et al. 2022a). Several plant-derived *α*-dioxygenases have been described and applied for the production of aliphatic aldehydes, *e.g.,* from cucumber (Galliard and Matthew 1976), tobacco (Kawasaki et al. 1998; Hamberg et al. 1999), rice (Koeduka et al. 2002; Kaehne et al. 2011), *Arabidopsis thaliana* (Hamberg et al. 1999; Liu et al. 2006), and algae (Kajiwara et al. 1989; Akakabe et al. 1999). More recently, *α*-dioxygenases were identified in the cyanobacteriae *Crocosphaera subtropica* (Hammer et al. 2020), *Calothrix parietina*, and *Leptolyngbya* sp. (Kim et al. 2022b).

**Fig. 1:**
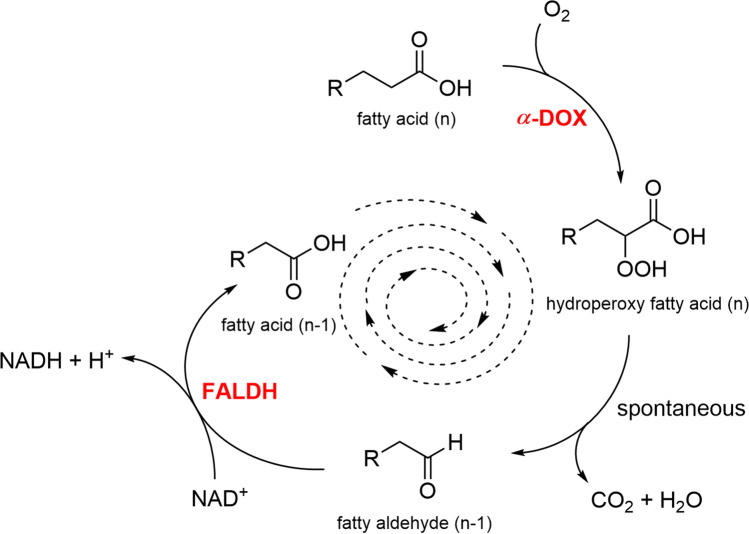
Catalytic cycle yielding chain-shortened fatty aldehydes and acids by oxidative decarboxylation via* α*-dioxygenase (*α*-DOX) and successive oxidation via fatty aldehyde dehydrogenase (FALDH)

The herein described *α*-dioxygenase reaction is naturally linked to a further oxidation of the aldehyde to the corresponding fatty acid by an aldehyde dehydrogenase (Fig. 1) (Hamberg et al. 2005). This reaction cycle was already assumed to be present in plants by Shine and Stumpf (1974) and is known to act as a defense mechanism against environmental stress and pathogen infections (Hamberg et al. 2002). The *α*-dioxygenase reaction needs oxygen as a co-substrate, while aldehyde dehydrogenases are usually dependent on NAD(P)^+^ (Buchhaupt et al. 2013). In the context of industrial biotechnology, there is a high demand for readily available and inexpensive substrates to raise profitability. Thus, naturally abundant materials with valuable contents are of significant interest. For the purpose of aldehyde biosynthesis, organisms rich in lipids might prove beneficial for the generation of complex aldehyde mixtures applying the biotechnological methods described.

Sea buckthorn (*Hippophae rhamnoides*) is a deciduous shrub distributed across Eurasia (Wang et al. 2014). The lipid fraction of sea buckthorn could serve as a promising candidate for aldehyde synthesis, since the pulp and seeds of the berries are relatively rich in lipids. The seeds are reported to contain up to 15% and the pulp up to 34% lipids in dry matter (Yang and Kallio 2002). Sea buckthorn seed oil mainly consists of polyunsaturated linoleic [18:2(9*Z*,12*Z*)] and *α*-linolenic acid [18:3(9*Z*,12*Z*, 15*Z*)], while the pulp’s predominant fatty acids are palmitic [16:0], palmitoleic [16:1(9*Z*)], oleic [18:1(9*Z*)], and linoleic acid [18:2(9*Z*,12*Z*)] (Yang and Kallio 2001). Sea buckthorn stands out for its high lipid contents and interesting fatty acid profile, wide distribution in nature, extreme temperature tolerance of –43 °C to + 40 °C, and drought resistance (Koskovac et al. 2017). These features make sea buckthorn oil an interesting candidate for industrial applications.

In this study, a repetitive tandem reaction of an *α*-dioxygenase from *Crocosphaera subtropica* (*Csα*-DOX) (Hammer et al. 2020) in combination with a fatty aldehyde dehydrogenase from *Vibrio harveyi* (*Vh*FALDH) (Buchhaupt et al. 2013) was developed to produce odorous carbon chain shortened fatty aldehydes from the corresponding fatty acids (Fig. 1). By using a combination of these two enzymes, it was possible to produce multiple different aldehydes with carbon chains shortened by one C-atom per reaction cycle in a one-pot reaction. The enzymatic tandem reaction demonstrated in the present work was optimized to obtain higher quantities of odor-active fatty aldehydes. Application of a lipid extract from sea buckthorn was chosen as an exemplary natural substrate for the reaction cycle. Upon lipid hydrolysis, the free fatty acids serve as substrates for the generation of complex odorous aldehyde mixtures that could be used as natural flavoring/fragrance ingredients. To identify the major aldehydes formed unambiguously, analytical standards of the most predominant fatty acids of sea buckthorn pulp oil were biotransformed and sensorially characterized. Molecular structures of the generated aldehydes in terms of the double-bond positions were verified, and the efficiency and substrate specificity of the process were estimated by semi-quantitation.

## Materials and methods

### Chemicals

Acetone (99.8%) and palmitoleic acid [16:1(9*Z*)] (99%) were obtained from Acros (Fair Lawn, NJ, USA). Nitrogen was purchased from Air Liquide (Düsseldorf, Germany). Decanal (96%), dodecanal (95%), and tridecanal (90%) were supplied by Alfa Aesar (Ward Hill, MA, USA). Coomassie Brilliant Blue R250 and sodium dodecyl sulfate (99%) were purchased from AppliChem (Darmstadt, Germany). Disodium hydrogen phosphate (99.5%), glycine (99%), imidazole (99.5%), lysogeny broth (LB) medium, potassium dihydrogen phosphate (98%), tris(hydroxymethyl)aminomethane (TRIS) (99%), and Triton X-100 were obtained from Carl Roth (Karlsruhe, Germany) and *iso*-octane from Merck (Darmstadt, Germany). Helium (5.0) was supplied by Praxair (Düsseldorf, Germany). Isopropyl *β*-D-1-thiogalactopyranoside (IPTG) (99%) and kanamycin sulfate (> 750 I.U./mg) were purchased from Serva (Heidelberg, Germany). 3-Acetylpyridine (98%), (*Z*)-7-decenal (97%), nicotinamide adenine dinucleotide (NAD^+^), oleic acid [18:1(9*Z*)] (99%), palmitic acid [16:0] (99%), and linoleic acid [18:1(9*Z*, 12*Z*)] (99%) were obtained from Sigma Aldrich (St. Louis, MO, USA). Heptadecanal (97%), hexadecanal (97%), (*Z*)-11-hexadecenal (95%), pentadecanal (97%), tetradecanal (95%), undecanal (97%), and decanal (97%) were purchased from TCI (Tokyo, Japan). Hydrochloric acid 25% (HCl) was obtained from Th. Geyer (Renningen, Germany). Acetonitrile (99.9%) was supplied by VWR Chemicals (Radnor, PA, USA).

### Enzymes, heterologous expression, and purification

A pETDuet vector with a codon-optimized gene insert encoding for *Vh*FALDH was produced by GENEART (Regensburg, Germany) (GenBank accession number: ON677428) (Buchhaupt et al. 2013). This gene was transferred to a pET28a-vector to add an N-terminal HIS-Tag using restriction enzymes *Nde*I and *Xho*I (Thermo Scientific) and T4-Ligase (Thermo Scientific). The construct was validated by sequencing (Eurofins, Luxemburg) using a T7-primer. *E. coli* W3110 (DE3) cells were transformed with this vector. Preparation of *Csα*-DOX was performed as described by Hammer et al. (2020) (GenBank accession number: ON711410). Both *E. coli* strains were cultivated in LB medium with 30 µg/mL kanamycin to an OD_600_ of 1.4–1.6 at 37 °C in baffled shake flasks. Induction was initiated by addition of isopropyl *β*-D-1-thiogalactopyranoside (IPTG) (0.5 mM). Expression was conducted at 21 °C (*Csα*-DOX) and 18 °C (*Vh*FALDH) overnight, after which the cells were harvested by centrifugation.

For enzyme purification, the cells were mixed with extraction buffer (25% (w/v), cell wet weight, 50 mM phosphate buffer (pH 7.5), 20 mM imidazole), and lyzed by a sonifier (Bandelin Sonopuls, Berlin, Germany). One percent of Triton X-100 was added to the buffer to enhance extraction performance. After centrifugation, the enzymes were purified by means of a nickel loaded nitrilotriacetic acid column (Ni–NTA) (Macherey–Nagel, Düren, Germany). The His-tagged enzymes were eluted with 50 mM phosphate buffer (pH 7.5) containing 250 mM imidazole, concentrated by centrifugal filter devices (Merck, Darmstadt, Germany) with molecular mass cutoff of 30 kDa for *Vh*FALDH and 50 kDa for *Csα*-DOX, and desalted using a PD-10 column (GE Healthcare, Buckinghamshire, UK).

Enzyme concentrations were determined after purification by photospectroscopy using an Implen NanoPhotometer^©^ P300 (Munich, Germany) with 5 µL sample volume. The specific ε values used were 34,295 L/(mol ∙ cm) for *Vh*FALDH (MW 56,649.95) and 96,433 L/(mol ∙ cm) for *Csα*-DOX (MW 69,894.92). The values were calculated based on the respective amino acid sequences using the ProtParam calculator of the Swiss Institute of Bioinformatics (Walker 2005).

Enzyme expression was checked by sodium dodecyl sulfate polyacrylamide gel electrophoresis (SDS-PAGE) (Laemmli 1970) with 4% stacking and 12% resolving gel and Coomassie R250 staining.

### Enzyme activity

To determine *Csα*-DOX activity, the consumption of oxygen in the reaction mixture was measured by use of an optical oxygen probe (Microx TX3, PreSens, Regensburg, Germany). The total reaction volume in the microtiter plate was 300 µL containing 5 mM dodecanoic acid as a fatty acid standard substrate and 10 µg/mL of *Csα*-DOX. The probe was calibrated with saturated sodium dithionite solution and with double distilled water, saturated with compressed air for at least 5 min. The measurements were performed in microtiter plates at 25 °C and 250 rpm stirrer speed every 10 s. The values from 20–120 s after addition of the enzyme solution were used for calculation. Fivefold determinations were conducted to calculate enzyme activity.

Since the reaction of *Vh*FALDH is NAD(P)^+^-dependent, enzyme activity was measured spectrophotometrically via absorbance of NAD(P)H. The measurements were performed in microtiter plates at 340 nm and 25 °C. The total volume per well was 200 µL containing 50 µM NAD^+^, 1 µg/mL *Vh*FALDH, and 50 µM undecanal were used. NAD^+^ was applied instead of NADP^+^ due to a higher enzyme activity observed in the presence of the former (supplementary Fig. S1). Triplicates were measured in each case. The extinction coefficient used was determined as 3328 L/(mol ∙ cm). The measurement was immediately started after addition of NAD^+^, and activity was determined within the first 60 s.

### Application of the coupled enzyme reaction

#### Method optimization with model substrate oleic acid

Biotransformation experiments were performed in 20 mL headspace vials (Th. Geyer, Renningen, Germany) containing 5 mg oleic acid, dispersed in 2 mL phosphate buffer (50 mM, pH 7.5) by means of an ultrasonic bath (Bandelin Sonorex, Berlin, Germany) for 5 min. Two hundred µL cofactor NAD^+^ (5 mM) and purified *Csα*-DOX and *Vh*FALDH enzyme solutions were added to a final reaction volume of 4 mL. ~ 20 glass beads (Ø 3 mm) were added to increase dispersion. Incubation was performed in a rotary shaker (40 rpm, Stuart Rotator SB3, Merck, Darmstadt, Germany) at 24 °C in the dark. Incubation time (1 h, 4 h, 8 h), enzyme ratios of *Csα*-DOX to *Vh*FALDH (4:1, 8:1, 12:1, and 16:1, where *Vh*FALDH was kept constant at a concentration corresponding to an activity of 12.5 U/L), and total enzyme activity with constant relative enzyme ratios of 8:1 (100:12.5 U/L, 50:6.25 U/L, 150:18.75 U/L) were compared. After incubation, the reaction mixtures were cooled in an ice bath and the reaction was stopped by addition of 200 µL of 4 M HCl.

Instrumental analysis was performed by means of HS–SPME–GC–MS–O. SPME extraction was executed with a polydimethylsiloxane/divinylbenzene (PDMS/DVB) coated fiber (1 cm length, 65 µm) (Merck, Darmstadt, Germany). Samples were incubated for 10 min at 60 °C and extracted for 30 min at 60 °C at 250 rpm agitation rate using a GERSTEL MPS2 XL autosampler (Mülheim/Ruhr, Germany). Analytes were desorbed in an SPME liner within the inlet of an Agilent (Waldbronn, Germany) A7890 GC system, equipped with an Agilent VF-WAXms column (30 m L × 0.25 mm ID × 0.25 µm film thickness) at 250 °C for 90 s and injected with a split ratio of 10:1. Helium (5.0) was used as a carrier gas with a constant flow rate of 1.56 mL/min. The oven was programmed with an initial temperature of 40 °C (3 min) and heating with 5 °C/min to 240 °C (12 min). Detection was performed with an Agilent 7000B triple quadrupole tandem mass spectrometer with the following parameters applied: electron ionization energy, 70 eV; ion source temp., 230 °C; scan range, *m*/*z* 33–300; quadrupoles temp., 150 °C. The GC system was equipped with a GERSTEL olfactory detection port ODP 3 (transfer line temp., 250 °C; mixing chamber temp., 150 °C; makeup gas, N_2_).

#### Biotransformation of sea buckthorn oil and standard fatty acids

Sea buckthorn pulp oil (obtained from Henry Lamotte Oils GmbH, Bremen, Germany) was enzymatically hydrolyzed. Therefore, 5 mg oil was dispersed in 2 mL phosphate buffer (50 mM, pH 7.5) by means of an ultrasonic bath for 5 min. Lipase (6 U, E.C. 3.1.1.3) from *Candida rugosa* (Sigma Aldrich) was added and the mixture was incubated in a rotary shaker (40 rpm) at 24 °C for 3 h in the dark.

Besides the hydrolyzed lipid extract, standard fatty acids palmitic, palmitoleic, oleic, and linoleic acid were prepared as indicated above. Biotransformation experiments were executed under optimized conditions. 150 U/L *Csα*-DOX and 18.75 U/L *Vh*FALDH were added to the substrate-cofactor dispersion. After incubation for 4 h, the reaction was stopped by addition of HCl as described above, and the samples were stored at –20 °C until analysis.

#### Compound identification and GC–olfactometry

HS–SPME–GC–MS analysis for determination of retention indices (van den Dool and Kratz 1963) and GC–MS–olfactometry were carried out as described under “Method optimization with model substrate oleic acid.” Olfactometric assessment was executed by two trained assessors.

Retention indices on a nonpolar GC column were determined after SPME extraction on an Agilent 7890B GC system equipped with an Agilent DB-5 ms column (30 m L × 0.25 mm ID × 0.25 µm film thickness). The flow rate of the carrier gas helium (5.0) was set to 1.2 mL/min (constant flow). The initial temperature was held at 40 °C for 3 min, heated to 320 °C with 5 °C/min and held for 12 min. Detection was performed with an Agilent 5977B mass spectrometer (electron ionization energy, 70 eV; ion source temp., 230 °C; quadrupole temp., 150 °C).

The reaction products were identified by comparison of mass spectra to those of the NIST MS library (NIST MS Search 2011, National Institute of Standards and Technology, Gaithersburg, MD, USA) and of retention indices calculated from nonpolar (DB-5 ms) and polar (VF-WAXms) GC columns with published retention indices or analyzed authentic standards.

For determination of double-bond positions, enzymatic reaction mixtures of palmitoleic, oleic, and linoleic acid as well as incubated blanks (either without substrates or enzymes) were extracted three times with 4 mL of *n*-pentane. The extracts were concentrated under a gentle stream of nitrogen to a volume of approximately 10 µL and diluted with 500 µL of acetonitrile. Five µL of the resulting solutions was mixed with 93 µL of acetonitrile and 1 µL of acetylpyridine and 1 µL of formic acid to allow for nano-electrospray ionization–online PB functionalization–tandem mass spectrometric experiments (nanoESI–online–PB–MS/MS). Nanospray capillaries (2 µm ID, in-house pulled; P-97, Sutter Instruments, Novato, CA, USA) were loaded with 10 µL of sample. A home-built nanoESI source with a voltage of 700 V between nanospray capillary and mass spectrometer inlet was applied. The emitting sample was exposed to the light of a low-pressure mercury UV lamp (254 nm emission maximum; UVP, Upland, CA, USA) as described previously (Esch and Heiles 2018). All measurements were performed with an orbital trapping mass spectrometer in positive-ion mode (Q Exactive™ HF-X, Thermo Scientific, San Jose, CA, USA), and higher energy collisional dissociation (HCD) experiments by employing 25 to 30 normalized collision energy (NCE) values.

#### Semi-quantitation

Saturated aldehydes with corresponding carbon chain lengths were used for semi-quantitation of not commercially available unsaturated fatty aldehydes. As a proof of concept for this approach, signal intensities of two saturated fatty aldehydes and available authentic unsaturated counterparts were compared by calculating relative response factors (supplementary Fig. S2).

Stock solutions of analytical standards of C_11_–C_18_ saturated aldehydes (~ 1 g/L dissolved in acetone) were diluted to a final concentration of 100 µg/L in 50 mM phosphate buffer (pH 7.5). Biotransformation samples were diluted 1:500 (linoleic acid: 1:100) in phosphate buffer (50 mM, pH 7.5) to a final volume of 4 mL and spiked with 200 µg/L (*Z*)-7-decenal as internal standard.

Instrumental analysis was performed by means of HS–SPME–GC–MS with nonpolar DB-5 ms column as stated above. Semi-quantitation of fatty aldehydes from biotransformation experiments was carried out with biological duplicates and triplicate analytical measurements.

## Results

### Establishment of the coupled enzyme reaction

Both enzymes were successfully expressed and purified to electrophoretic homogeneity. The enzymatic tandem reaction was successfully applied for the biotransformation of oleic acid as a model substrate. In order to increase the yield of the target aldehydes and extend the number of reaction cycles, the parameters incubation time, enzyme ratio, and concentration were optimized.

**Fig. 2:**
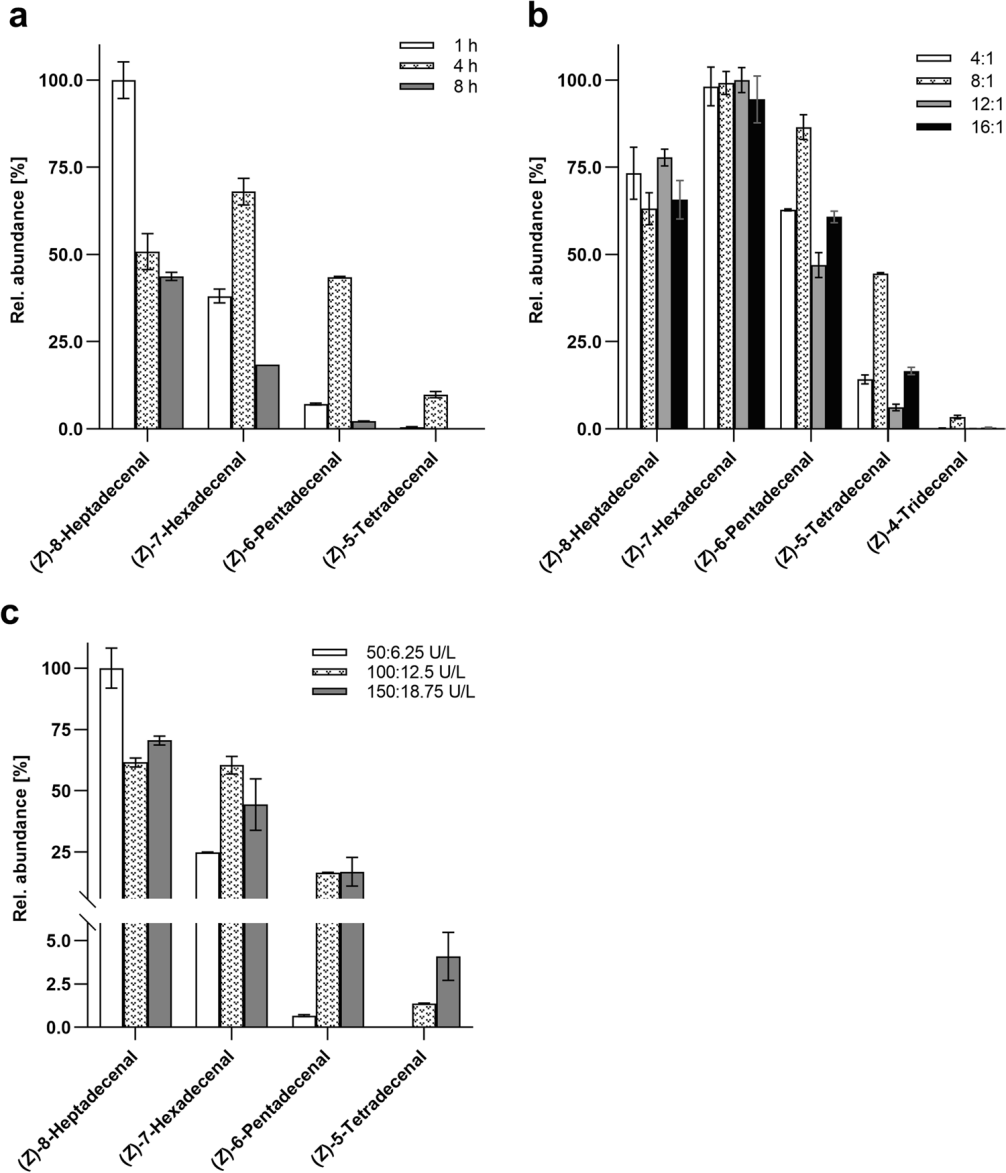
Optimization of biotransformation: **a** Comparison of incubation times, **b** enzyme unit ratios *Csα*-DOX:*Vh*FALDH [U/L] (4:1 = 50:12.5, 8:1 = 100:12.5, 12:1 = 150:12.5, 16:1 = 200:12.5), **c** total enzyme activity concentrations. Error bars indicate standard deviations

Biotransformation mixtures incubated for 1 h showed the highest signal intensity for the primary reaction product (*Z*)-8-heptadecenal. However, metabolites of further reaction cycles were less abundant in comparison with longer incubation times (Fig. 2a). While incubation for 1 h and 8 h resulted in 3 reaction cycles, however samples incubated for 4 h showed a 4^th^ cycle exhibiting a remarkably high signal intensity for (*Z*)-5-tetradecenal and even minor amounts of (*Z*)-4-tridecenal resulting from a 5^th^ reaction cycle. With prolonged incubation, the activity of the *Csα*-DOX decreased compared to that of *Vh*FALDH, leading to reduced aldehyde concentrations.

A *Csα*-DOX to *Vh*FALDH ratio of 8:1 (corresponding to 100:12.5 U/L) was found to be most efficient (Fig. 2b). With this ratio, 5 reaction cycles were observed, while other ratios resulted in a maximum of 4 cycles and significantly lower signal intensities of the aldehydes (*Z*)-6-pentadecenal (3^rd^ cycle) and (*Z*)-5-tetradecenal (4^th^ cycle). With the optimized parameters of 8:1 enzyme ratio and 4 h incubation time, total enzyme activity was varied to investigate effects on cycle number and aldehyde yields (Fig. 2c). Reduced enzyme concentrations, corresponding to 50 U/L *Csα*-DOX and 6.25 U/L *Vh*FALDH, showed an insufficient efficacy regarding the reaction cycle numbers. Conversely, the concentration of the 1^st^ cycle reaction product (*Z*)-7-heptadecenal was higher in comparison with higher doses applied. An increase of enzyme concentration by 50% (150:18.75 U/L) resulted in twofold abundance of 4^th^ cycle product (*Z*)-5-tetradecenal in comparison with initial concentration.

### Production of fatty aldehydes

#### Biotransformation of sea buckthorn oil

The biotransformation of hydrolyzed sea buckthorn oil resulted in the generation of numerous fatty aldehydes (Table 1, supplementary Fig. S3). In order to investigate the substrate spectrum in correlation to the aldehydes generated, the fatty acid profile of sea buckthorn oil was determined (Table S1). The most abundant fatty acids were palmitic, palmitoleic, and oleic acid. In smaller amounts, vaccenic, linoleic, linolenic, and stearic acid were detected. GC–MS–O analyses demonstrated that most of the aldehydes formed by biotransformation of hydrolyzed sea buckthorn oil were olfactorily perceived with manifold odor impressions (Table 1). The most abundant odor attributes were soapy, waxy, and green. Furthermore, citrus-like, metallic, and fatty odors were detected. Upon biotransformation, a total yield of ~ 203 mg aldehydes per gram employed oil was obtained. Pentadecanal and (*Z*)-8-pentadecenal were the most abundant fatty aldehydes, followed by (*Z*)-8-heptadecenal, tetradecanal, and (*Z*)-7-tetradecenal.

**Table 1:** Products of the biotransformation of hydrolyzed sea buckthorn oil with retention indices, odor impressions, and approximated yields (mg aldehyde per g lipid extract). Retention indices in parentheses are calculated from aldehydes obtained upon biotransformation of single fatty acids (cf. Tables 2, 3, 4, and 5) or from literature, whenever a reference is cited. Errors are given as standard deviations. References: a — Choi (2005), b — Choi (2006), c — Eyres et al. (2005), d — Chisholm et al. (2003), e — Marques et al. (2000), f — Miyazawa et al. (2007), g — Kajiwara et al. (1989), h — Hamberg et al. (1999)

Retention index		Compound	Odor impression		Probable precursor [number of cycles]	Yield [mg/g]
DB-5 ms	VF-WAXms		GC–O	Literature		
1408 (1408)	1703 (1705)	dodecanal	green, citrus, waxy	green^a^, citronellol-like^c^	16:0 [4]	0.4 ± < 0.1
1510 (1511)	1810 (1811)	tridecanal	green, waxy, soapy, textile	fresh, green^a^	16:0 [3]	1.9 ± 0.3
1491 (1491)	1843 (1842)	(*Z*)-6-tridecenal	soapy, waxy	n.r	16:1(9*Z*) [3]	0.2 ± 0.0
1614 (1613)	1917 (1919)	tetradecanal	green, soapy, floral, citrus	fresh, herbaceous^a^	16:0 [2]	10.6 ± 1.6
1593 (1591)	1947 (1948)	(*Z*)-5/**7**-tetradecenal*	soapy, fresh, green, grapefruit	(5*Z*): soapy, green^d^	18:1(9*Z*) [4]/ **16:1(9*Z*) [2]**/ 18:1(11*Z*) [4]	7 ± 1
1722 (1717)	2025 (2024)	pentadecanal	soapy, waxy, brothy	pungent, spicy, woody^c^	16:0 [1]	35.8 ± 5.2
1697 (1693)	2052 (2053)	(*Z*)-6/**8**-pentadecenal*	green, herbaceous, metallic	n.r	**16:1(9*Z*) [1]**/ 18:1(11*Z*) [3]	21 ± 2
1793 (1792)	2139 (2139)	(*Z*)-7-hexadecenal		n.r	18:1(9*Z*) [2]	2 ± 0
1795 (1800)^e^	2142 (2147)^e^	(*Z*)-9-hexadecenal		n.r	18:1(11*Z*) [2]	1 ± 0
1785 (1785)	2220 (2225)	(*Z,Z*)-7,10-hexadecadienal	waxy, fatty	n.r	18:2(9*Z*,12*Z*) [2]	0.7 ± 0.1
1918 (1919)^f^	2234 (2221)^f^	heptadecanal		sweet^b^	18:0 [1]	2.3 ± 0.4
1897 (1894)	2240 (2238)	(*Z*)-8-heptadecenal		n.r	18:1(9*Z*) [1]	83 ± 6
1900	2242	(*Z*)-10-heptadecenal		n.r	18:1(11*Z*) [1]	27 ± 3
1886 (1887)	2330 (2330)	(*Z,Z*)-8,11-heptadecadienal	fatty, brothy, tallow	green, algae-like^g^	18:2(9*Z*,12*Z*) [1]	10 ± 1
n.d (1895)^f^	2373 (2358)^f^	(*Z,Z,Z*)-8,11,14-heptadecatrienal	fatty, waxy	seaweed^h^	18:2(9*Z*,12*Z*,15*Z*) [1]	1 ± 0

n.r. — not reported, n.d — not detected

Unsaturated compounds were semi-quantified using authentic saturated homologues (cf. supplementary Fig. S2 for general validation of this procedure). Results for these compounds are therefore only given without decimal digits, if larger than 1

^*^ Co-eluting compounds. Double-bond position marked in **bold** is considered as expected main constituent/precursor

#### Biotransformation of single fatty acids

**Fig. 3:**
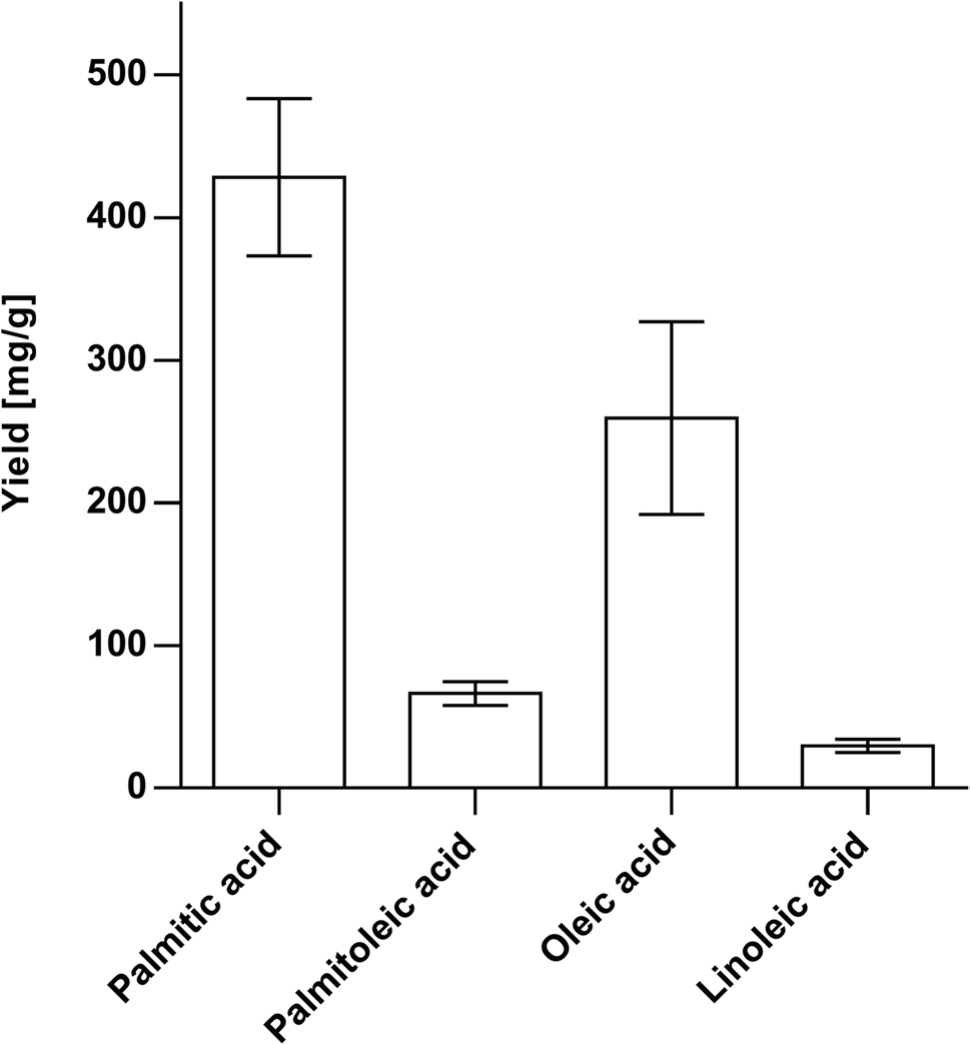
Yield of aldehydes per gram substrate supplemented for biotransformation experiments with *Csα*-DOX and *Vh*FALDH. Error bars indicate standard deviations

GC–MS–O analyses of biotransformed standard fatty acids revealed a wide variety of odor impressions within the formed fatty aldehydes (Table 2, 3, 4 and 5). Odors described as soapy, waxy, and green were most abundant and not clearly limited to a specific structural feature. However, a tendency of fatty odor perception with increase in chain length could be observed. Results of semi-quantitation (Fig. 3) clearly demonstrated a substrate specificity toward saturated fatty acid palmitic acid, which showed the highest total yield with roughly 430 mg aldehyde per gram substrate. The biotransformation of unsaturated fatty acids resulted in significantly lower yields, with oleic acid yielding the highest aldehyde concentrations of ~ 260 mg/g, followed by palmitoleic acid with ~ 67 mg/g. The conversion of polyunsaturated linoleic acid was even lower with a yield of ~ 30 mg/g.

**Table 2:** Products of the biotransformation of palmitic acid [16:0] with retention indices, odor impressions, and yields. Retention indices in parentheses are from cited literature. Errors are given as standard deviations. References: a — Chisholm et al. (2003); b — Mahattanatawee et al. (2005); c — Choi (2005); d — Sukhonthara et al. (2009); e — Eyres et al. (2005)

Retention index		Compound	Odor impression		Yield [mg/g]
DB-5 ms	VF-WAXms		GC–O	Literature	
1307 (1307)^d^	1598 (1610)^a^	undecanal	citrus, soapy, fresh, metallic	citrus^a^, fruity, floral, spicy^e^	0.3 ± 0.0
1408 (1401)^c^	1705 (1718)^c^	dodecanal	waxy, soapy, green, coriander	herbaceous, waxy^a^, soapy^b^, green^c^, pungent, spicy, floral, citronellol-like^e^	2.4 ± 0.4
1511 (1503)^c^	1811 (1824)^c^	tridecanal	green, soapy, grapefruit	fresh, green^c^	8.9 ± 1.2
1613 (1613)^d^	1919 (1924)^c^	tetradecanal	soapy, fatty, metallic	fresh, herbaceous^c^	100.7 ± 12.2
1717 (1710)^d^	2024 (2030)^d^	pentadecanal	waxy	pungent, spicy, woody^e^	315.9 ± 41.3

#### Compound identification

**Fig. 4:**
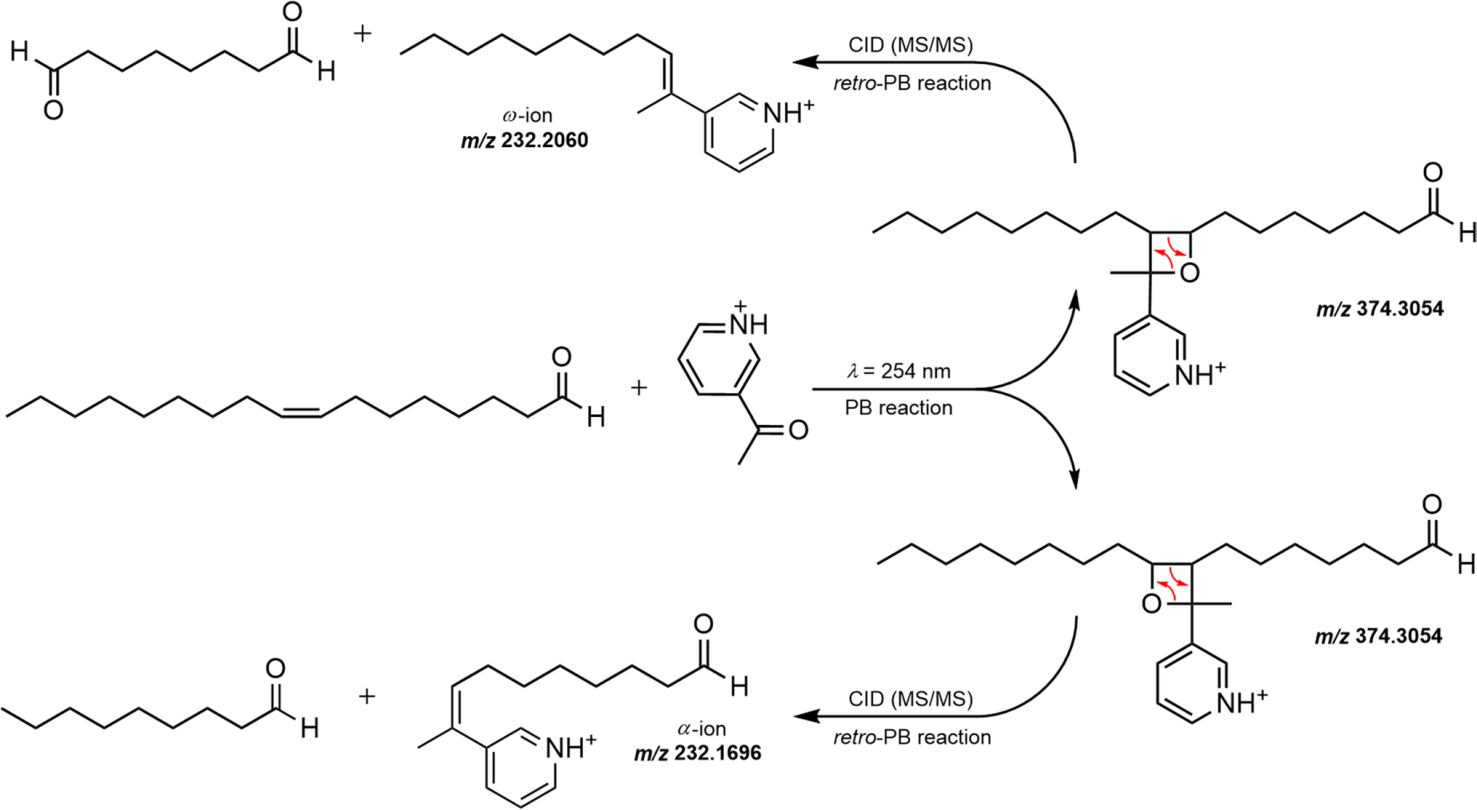
Exemplary PB reaction, of the analyte (*Z*)-8-heptadecenal with 3-acetylpyridine. HCD experiments initiated fragmentation of PB-products yielding diagnostic *α*- and *ω*-ions confirming the double-bond position of the aldehyde between C_8_ and C_9_

Fatty aldehydes formed during biotransformation via the proposed enzyme tandem reaction were tentatively identified by means of MS data and retention indices, calculated from analyses on polar and nonpolar GC columns and comparison to published data. As several compounds had not been reported previously, structure elucidation was performed via PB reaction (Fig. 4). The observed MS signals indicated the successful generation of adducts of 3-acetylpyridine and a series of expected unsaturated fatty acids and aldehydes for all three samples. The reaction mixture of oleic acid showed *m/z* values of 404, 390, 376, 374, 362, 360, 348, 346, 334, 332, and 318. For instance, *m/z* 374 resulted from the two isomeric oxetanes (PB products) of heptadecanal and 3-acetylpyridine. Retro-PB reaction, initiated during HCD experiments, revealed the double-bond position between C_8_ and C_9_, as characteristic fragments of *m/z* 232.1696 (*α*-ion) and 232.2060 (*ω*-ion) were detected. On the other hand, PB adducts with *m/z* 376, 362, 348, 346, 334, 332, 320, 318, and 304 were detected in the palmitoleic acid, *m/z* 402, 388, 374, 372, 360, 358, 346, 344, and 330 in the linoleic acid sample. Diagnostic fragmentation signals of all PB products are reported in supplementary Tables S2–S4. While *ω*-ions were always detectable, signals of *α*-ions were found only in some cases. Apart from not transformed substrate acids, blanks contained no or more than 1000-fold lower signal intensities of the relevant *m/z* values.

## Discussion

In the current study, a biotechnological approach yielding numerous targeted odor-active fatty aldehydes with promising efficiency was developed. Enzyme activity and resulting coupled reaction of *Csα*-DOX and *Vh*FALDH were shown to be well suitable for the efficient production of targeted fatty aldehydes from various long-chain fatty acids.

The efficacy of fatty aldehyde generation was optimized by varying reaction parameters by use of oleic acid as a model substrate. One of the main challenges when dealing with coupled enzyme reactions in a one-pot bioprocess is to assure the *quasi* co-working of enzymes in order to maximize the yield of target compounds. Since enzyme activities of the applied enzymes are likely to differ from each other over time, the generation of aldehydes was investigated for varying incubation times. The highest signal intensities were obtained after 4 h. Shorter incubation resulted in the highest concentration of the C_n-1_ aldehyde (*Z*)-7-heptadecenal, whereas product yields of the following reaction cycles were considerably lower. For targeted applications in industrial processes, the incubation time might serve as powerful tool for controlling the product pattern, and thus overall odor impression of the resulting aldehyde mixture. However, with prolonged incubation times of more than 4 h, a decrease of total aldehyde yield was observed. A lower enzyme stability of *Csα*-DOX might well explain this observation. As a result, an excess *Vh*FALDH activity catalyzes the oxidation of aldehydes as the terminal reaction step. The optimal enzyme activity ratio of *Csα*-DOX and *Vh*FALDH was thus crucial for the purpose of fatty aldehyde generation. Throughout biotransformation, *Csα*-DOX should exceed *Vh*FALDH activity to avoid augmented oxidation of aldehydes to acids. An activity ratio of 8:1 (*Csα*-DOX:*Vh*FALDH) was identified as most suitable for generating an excess of fatty aldehydes. The effect of total enzyme concentration in the reaction mixture on aldehyde production is an important parameter, especially when it comes to bioprocess upscaling to an industrial scale. Hence, varying total enzyme concentrations were applied for biotransformation trials. The results of laboratory scale experiments revealed that increased enzyme concentrations resulted in higher aldehyde yields, thus no needless excess of enzyme introduction was observed. Nevertheless, results indicated that varying enzyme concentrations could serve as a tool for regulation of aldehyde distribution in the resulting reaction mixture, *e.g.,* if C_n-1_ aldehyde was aimed as predominant target compound, a lower enzyme concentration would be most suitable.

The optimized enzymatic tandem reaction was successfully applied for biotransformation of a hydrolyzed lipid extract from sea buckthorn pulp and single aliphatic fatty acids yielding odor-active fatty aldehydes. The fatty acid profile of sea buckthorn oil was in accordance with published data (Yang and Kallio 2001; Zielińska and Nowak 2017). The predominant fatty acids palmitoleic, oleic, and linoleic acid are of particular interest, since their chain-shortened fatty aldehydes produced via the coupled enzyme reaction have been scarcely described as aroma ingredients and their chemical synthesis is highly complex. GC–MS–O analyses revealed most of them being odor-active, exhibiting even auspicious smells. Most of the *Z*-unsaturated aldehydes have not yet been described in terms of their odorant properties.

Biotransformation of the most abundant fatty acids present in sea buckthorn pulp oil indicated a clear substrate preference toward the saturated fatty acid palmitic acid in comparison with unsaturated substrates. Earlier studies on the substrate specificity of the well described *α*-dioxygenase from *Oryza sativa* (*Osα*-DOX) did not show a clear preference for palmitic acid compared to unsaturated oleic or palmitoleic acids (Koszelak-Rosenblum et al. 2008). Furthermore, investigations on *Arabidopsis thaliana α*-dioxygenase (*Athα*-DOX) even showed opposite results with substrate preference toward unsaturated oleic and palmitoleic acid compared to saturated stearic and palmitic acid (Liu et al. 2006; Koszelak-Rosenblum et al. 2008). Surprisingly, the biotransformation of the polyunsaturated linoleic acid resulted in lowest yields of aldehydes. This likely results from the steric hindrance caused by the two *Z*-configured double bonds, which might prevent a proper docking of the ligand into the active site. However, this seems to be quite specific for the herein applied *Csα*-DOX since earlier studies on *α*-dioxygenases of various other organisms showed a high acceptance toward linoleic acid (Kajiwara et al. 1989; Koeduka et al. 2002; Koszelak-Rosenblum et al. 2008; Bannenberg et al. 2009). Recently, a study on the substrate specificity of a cyanobacterial *α*-dioxygenase (Kim et al. 2022b) showed results that are in good agreement with our findings. Thus, low substrate acceptance for polyunsaturated fatty acids is possibly a cyanobacteria-specific trait. Interestingly, palmitoleic acid gave lower aldehyde yields in comparison with oleic acid even though the double bond of both is located at C_9_. The coupled enzyme reaction terminated when reaching the cycle with products exhibiting a double bond at C_5_. Nevertheless, for oleic acid a 5^th^ reaction cycle, yielding a product with the unsaturated bond at C_4_, could be observed. The termination at these positions could be a result of steric hindrance within the binding site of the enzymes. This would be in accordance with findings that indicated a drastic decrease of *Osα*-DOX activity for substrates with unsaturated bonds within the first 6 carbon atoms (Koszelak-Rosenblum et al. 2008). Further investigations suggest that the first 7 carbon atoms are pivotal for substrate binding within the active site of *Osα*-DOX and related *Athα*-DOX, and therefore for an efficient catalysis (Goulah et al. 2013).

To verify the structural properties of reaction products, mass spectrometric fragmentation analyses of PB photoproducts of unsaturated compounds enabled the determination of their double-bond positions. This targeted approach renders elaborate purification procedures unnecessary and allows for an investigation of different compounds in parallel. Typically, it is employed for the characterization of different types of lipids (Esch and Heiles 2018; Wäldchen et al. 2019), but was already applied for structure elucidation in flavor research as well (Birk et al. 2019). By means of nanoESI–online–PB–MS/MS experiments, unsaturated positions of all fatty aldehydes, assigned in (Tables 3, 4, and 5), and their intermediate fatty acids were verified (see Supplementary Tables S2–S4 for assignments of all *m/z* signals and fragmentation). While the applied SPME–GC–MS technique was well suitable for the analysis of volatile fatty aldehydes, the corresponding fatty acids could not be analyzed simultaneously due to their high boiling points as well as inefficient desorption resulting in persistent memory effects on the SPME fibers. Thus, PB-MS/MS was also employed to unambiguously prove the presence of fatty acids.

**Table 3:** Products of the biotransformation of palmitoleic acid [16:1(9*Z*)] with retention indices, odor impressions, and approximated yields. Retention indices in parentheses are from cited literature. Errors are given as standard deviations. References: a — Chisholm et al. (2003); b — Wakamura et al. (1999)

Retention index		Compound	Odor impression		Yield [mg/g]
DB-5 ms	VF-WAXms		GC–O	Literature	
1388 (1364)^a^	1749 (1753)^a^	(*Z*)-5-dodecenal	waxy, musty	piney, waxy^a^	< 0.1
1491 (1471)^a^	1842	(*Z*)-6-tridecenal	waxy, green	n.r	0.2 ± 0.0
1591 (1609)^a^	1948 (1962)^b^	(*Z*)-7-tetradecenal	soapy, coriander	n.r	15 ± 2
1693	2053	(*Z*)-8-pentadecenal	green, fatty, chicken	n.r	52 ± 6

n.r. — not reported

Unsaturated compounds were semi-quantified using authentic saturated homologues

**Table 4:** Products of the biotransformation of oleic acid [18:1(9*Z*)] with retention indices, odor impressions, and approximated yields. Retention indices in parentheses are from cited literature. Errors are given as standard deviations. References: a — Chisholm et al. (2003); b — Wakamura et al. (1999)

Retention index		Compound	Odor impression		Yield [mg/g]
DB-5 ms	VF-WAXms		GC–O	Literature	
1492	1841	(*Z*)-4-tridecenal	n.d	n.r	0.3 ± 0.1
1590 (1565)^a^	1948 (1962)^a^	(*Z*)-5-tetradecenal	soapy, waxy	soapy, green^a^	3 ± 1
1692	2043	(*Z*)-6-pentadecenal	soapy, waxy	n.r	27 ± 9
1792 (1798)^b^	2139 (2144)^b^	(*Z*)-7-hexadecenal	waxy	n.r	83 ± 24
1894	2238	(*Z*)-8-heptadecenal	waxy	n.r	147 ± 33

n.r. — not reported, n.d. — not detected

Unsaturated compounds were semi-quantified using authentic saturated homologues

**Table 5:** Products of the biotransformation of linoleic acid [18:2(9*Z*,12*Z*)] with retention indices, odor impressions, and approximated yields. Retention indices in parentheses are from cited literature. Errors are given as standard deviations. References: a — Xu et al. (2020); b — Miyazawa et al. (2007); c — Kajiwara et al. (1989)

Retention index		Compound	Odor impression		Yield [mg/g]
DB-5 ms	VF-WAXms		GC–O	Literature	
1580	2019	(*Z,Z*)-5,8-tetradecadienal	sweet, waxy, citrus	n.r	0.3 ± 0.1
1683	2125	(*Z,Z*)-6,9-pentadecadienal	sweet, waxy, fatty	n.r	3 ± 0.6
1785	2225 (2225)^a^	(*Z,Z*)-7,10-hexadecadienal	soapy, herbaceous, spicy	n.r	11 ± 2
1887 (1889)^b^	2330	(*Z,Z*)-8,11-heptadecadienal	soapy, herbaceous, fatty, tallowy	green, algae-like^c^	16 ± 5

n.r. — not reported

Unsaturated compounds were semi-quantified using authentic saturated homologues

In the literature, *Z*-unsaturated fatty aldehydes are predominantly discussed in the context of insect pheromones (Swedenborg and Jones 1992; Teal et al. 1995; Silva et al. 2016; Becher et al. 2018) and to a lesser extent in the field of flavors and fragrances (Shi et al. 2013; Lorber et al. 2018). Odor impressions of numerous unsaturated fatty aldehydes like (*Z*)-6-tridecenal, (*Z*)-7-tetradecenal, and (*Z*)-8-heptadecenal or dienals like (*Z,Z*)-5,8-tetradecadienal, (*Z,Z*)-6,9-pentadecadienal, and (*Z,Z*)-7,10-hexadecadienal are described here for the first time (Tables 3, 4, and 5). Only few of the produced aldehydes have already been sensorily characterized (Kajiwara et al. 1989; Wakamura et al. 1999; Chisholm et al. 2003; Choi 2005; Eyres et al. 2005). The majority of the aldehydes showed  pleasant odor impressions with fresh, green, and soapy notes, which are of great interest to the flavor and fragrance industry. By subjecting naturally occurring oils and fats—such as the herein applied buckthorn-derived oil—to the developed biotechnological approach, the production of appealing aroma mixtures seems possible. Moreover, this approach includes solely NAD^+^ as a cofactor, which is relatively inexpensive, in comparison with cofactors such as NADH or NADPH. However, addition of stoichiometric amounts of cofactors to the reaction lacks economic efficiency. Hence, to increase the total turnover number, a suitable cofactor recycling system would be crucial for an efficient large-scale production approach. Therefore, the introduction of a NADH oxidase (E.C 1.6.99), *e.g.,* isolated from *Lactobacillus brevis* as proposed by Geueke et al. (2003) or applications of advanced enzyme immobilization techniques (Twala et al. 2012) are likely suitable for the herein described approach due to similar reaction conditions. In particular, immobilized enzymes would be of special interest for industrial processes due to their higher stability, re-usability and easy handling (Sheldon 2007). However, since NADH oxidases require molecular oxygen as electron acceptor, additional gassing would have to be considered in the experimental design.

The coupled one-pot enzyme reaction process with *Csα*-DOX and *Vh*FALDH was shown to be highly efficient to produce various homologous series of fatty aldehydes from the corresponding long-chain fatty acids. Gas chromatography–olfactometric analyses provided deeper insights into previously not reported odor impressions of *Z*-unsaturated fatty aldehydes. Aldehyde mixtures obtained from the biotransformation of buckthorn pulp or other oils could be of great interest to the food and cosmetic industry as flavorings or fragrances and represent a promising start for further screening of natural lipid extracts as substrates for bioprocess developments.

## Supplementary Information

Below is the link to the electronic supplementary material.

Supplementary file1 (PDF 321 KB)

## Data Availability

The datasets generated during and/or analyzed during the current study are available from the corresponding author on reasonable request.
