# Supplementary material for: An enzymatic tandem reaction to produce odor-active fatty aldehydes
Source: Appl Microbiol Biotechnol. 2022 Aug 30;106(18):6095–107. doi: 10.1007/s00253-022-12134-3 (PMC9468042; doi:10.1007/s00253-022-12134-3)
Supplement: Supplementary file 1 — Supplementary file1 (PDF 321 KB) [file 253_2022_12134_MOESM1_ESM.pdf]

Supplementary Information

An enzymatic tandem reaction to produce odor-active fatty aldehydes

Applied Microbiology and Biotechnology

Jean-Philippe Kanter[1], Philipp Jakob Honold[1], David Lüke[2], Sven Heiles[2], Bernhard Spengler[2], Marco Alexander Fraatz[1,3], Christoph Harms[4], Jakob Peter Ley[4], Holger Zorn[1,3], Andreas Klaus Hammer[1,3*]

[1] Institute of Food Science and Food Biotechnology, Justus Liebig University Giessen, Heinrich-Buff-Ring 17, 35392 Giessen, Germany

[2] Institute of Inorganic and Analytical Chemistry, Justus Liebig University Giessen, Heinrich-Buff-Ring 17, 35392 Giessen, Germany

[3] Fraunhofer Institute for Molecular Biology and Applied Ecology, Ohlebergsweg 12, 35394 Giessen, Germany

[4] Symrise AG, Muehlenfeldstrasse 1, 37603 Holzminden, Germany

* Corresponding author:

Andreas Klaus Hammer: Tel.: +49 (0) 641 972 19309, E-mail address: Andreas.Hammer@ime.fraunhofer.de

ORCIDs

Jean-Philippe Kanter	0000-0002-0596-8980
David Lüke	0000-0001-5351-920X
Sven Heiles	0000-0003-3779-8071
Bernhard Spengler	0000-0003-0179-5653
Marco Alexander Fraatz	0000-0002-5028-9653
Christoph Harms	0000-0002-6609-0358
Jakob Peter Ley	0000-0001-9388-4260
Holger Zorn	0000-0002-8383-8196
Andreas Klaus Hammer	0000-0001-9226-1993

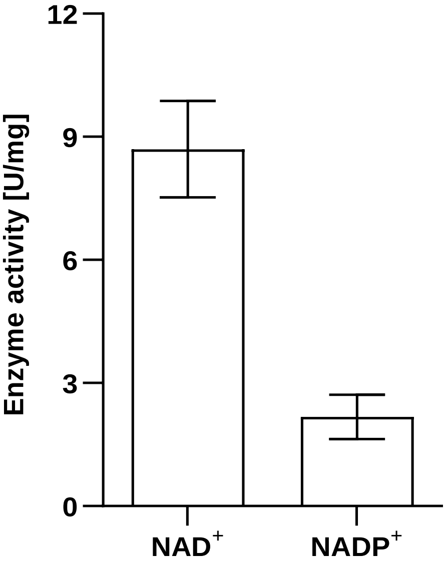

Fig. S1 Comparison of enzyme activities of *Vh*FALDH with cofactors NAD^+ *vs.* $NADP^+$. Error bars indicate standard deviations (GraphPad Prism 8)

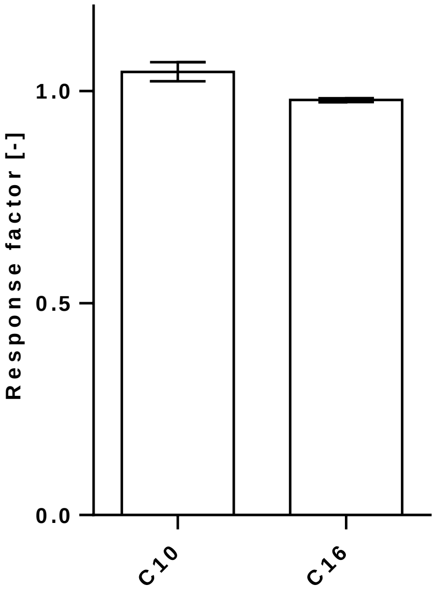

Fig. S2 Relative response factors of two commercially available unsaturated fatty aldehydes and their corresponding saturated counterparts. C10 depicts (*Z*)-7-decenal and decanal, C16 (*Z*)-11-hexadecenal and hexadecanal. Error bars indicate standard deviations. Results showed response factors of ~ 1.0 which indicated a marginal difference of mass spectrometric response of saturated and *Z*-unsaturated aldehydes of the same carbon chain lengths. Thus, saturated counterparts were considered as suitable for approximate quantitation of *Z*-unsaturated aldehydes (GraphPad Prism 8)

Table S1 Fatty acid composition of sea buckthorn pulp oil was determined by means of fatty acid methyl ester derivatization and subsequent instrumental analysis in terms of GC–MS according to the method described by Hammer et al. (2021)

no.	fatty acid (as fatty acid methyl ester)	relative share [%]
1	14:0	0.3
2	14:1(9*Z*)[a]	< 0.1
3	14:1(11*Z*)[a]	< 0.1
4	15:0	< 0.1
5	16:0	32.3
6	16:1(9*Z*)	25.9
7	16:1(11*Z*)	0.3
8	16:2(9*Z*,12*Z*)	< 0.1
9	17:0	< 0.1
10	17:1(9*Z*)	0.1
11	18:0	1.2
12	18:1(9*Z*)	25.0
13	18:1(11*Z*)	9.9
14	18:2(9*Z*,12*Z*)	3.2
15	18:3(9*Z*,12*Z*,15*Z*)	1.0
16	20:0	0.39
17	20:1(11*Z*)	0.2
18	22:0[a]	< 0.1

[a] identity not unambiguously clarified due to limited validity of mass spectrometric fragmentation pattern.

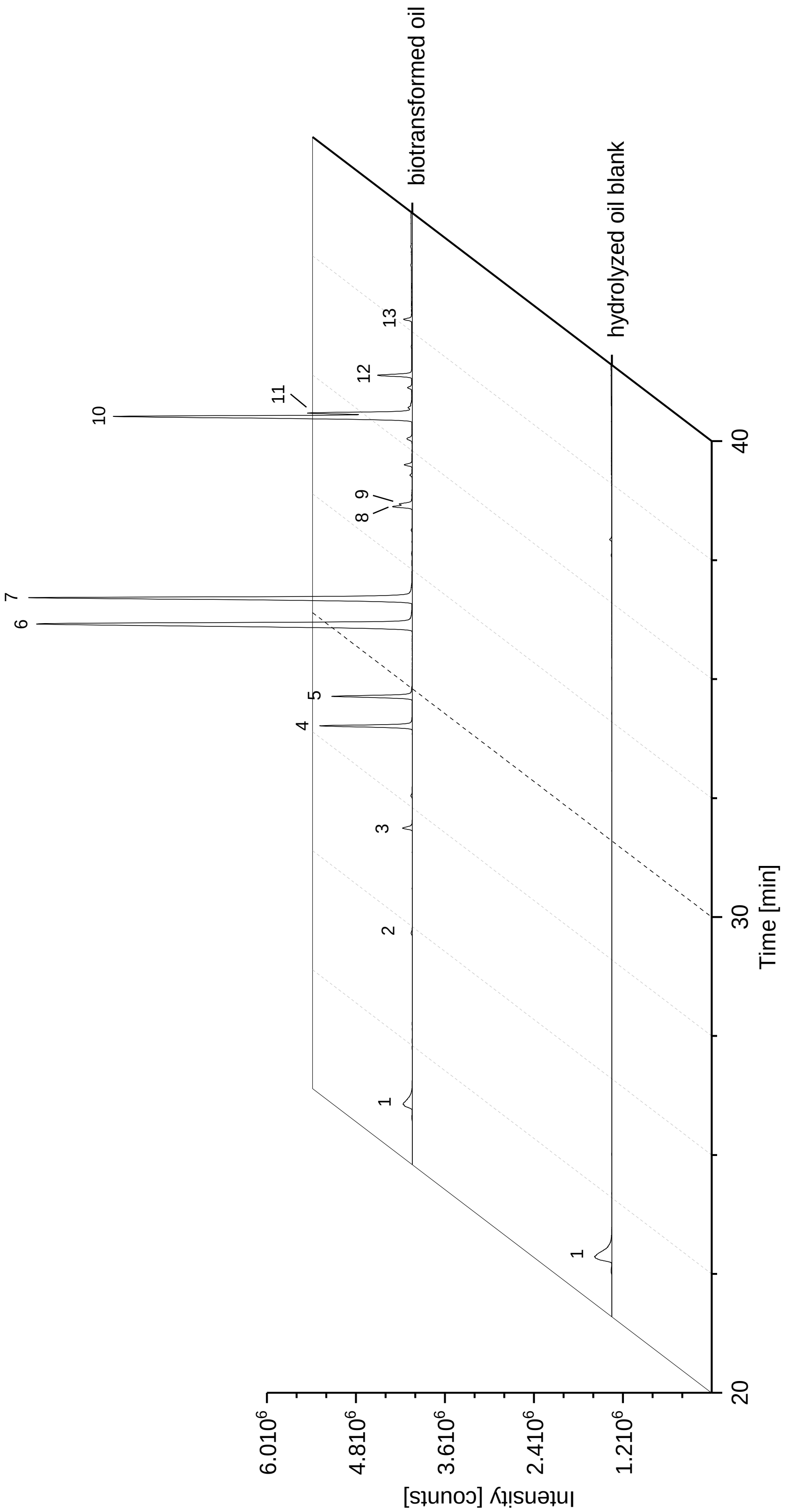

Fig. S3 GC–MS chromatograms of sea buckthorn pulp oil biotransformed with *Csα*-DOX and *Vh*FALDH (top) and corresponding non-biotransformed oil (lipase treated). **1** internal standard (*Z*)-7-decenal; **2** dodecanal; **3** tridecanal; **4** tetradecanal; **5** (*Z*)-5-tetradecenal + (*Z*)-7-tetradecenal; **6** pentadecanal; **7** (*Z*)-5-pentadecenal + (*Z*)-8-pentadecenal; **8** (*Z*)-7-hexadecenal; **9** (*Z*)-9-hexadecenal; **10** (*Z*)-8-heptadecenal; **11** (*Z*)-10-heptadecenal; **12** (*Z*,*Z*)-8,11-heptadecadienal; **13** (*Z*,*Z*,*Z*)-8,11,14-heptadecatrienal (OriginPro 2022)

Table S2 Identified Paternó-Büchi (PB) oxetane adducts along with their deduced diagnostic fragments (*α*- and *ω*-ions) during nanoESI–online–PB–MS/MS–analysis of the reaction mixture of oleic acid (see chapter “Identification”). All detected *m/z* signals are assigned in **bold** and showed a maximum deviation of ±5 ppm from theoretical values

compound	*m/z* [PB adduct]	*m/z* [*α*-ion]	*m/z* [*ω*-ion]
octadec-9-enoic acid	**404.3159**	262.1802	**232.2060**
heptadec-8-enoic acid	**390.3003**	248.1645	**232.2060**
hexadec-7-enoic acid	**376.2846**	234.1489	**232.2060**
pentadec-6-enoic acid	**362.2690**	220.1332	**232.2060**
tetradec-5-enoic acid	**348.2533**	206.1176	**232.2060**
tridec-4-enoic acid	**334.2377**	192.1019	**232.2060**
heptadec-8-enal	**374.3054**	**232.1696**	**232.2060**
hexadec-7-enal	**360.2897**	218.1539	**232.2060**
pentadec-6-enal	**346.2741**	204.1383	**232.2060**
tetradec-5-enal	**332.2584**	190.1226	**232.2060**
tridec-4-enal	**318.2428**	176.1070	**232.2060**

Table S3 Identified PB oxetane adducts along with their deduced diagnostic fragments (*α*- and *ω*-ions) during nanoESI–online–PB–MS/MS–analysis of the reaction mixture of linoleic acid (see chapter “Identification”). All detected *m/z* signals are assigned in **bold** and showed a maximum deviation of ± 5 ppm from theoretical values

compound	*m/z* [PB adduct]	*m/z* [*α*-ion 1]	*m/z* [*α*-ion 2]	*m/z* [*ω*-ion 1]	*m/z* [*ω*-ion 2]
octadeca-9,12-dienoic acid	**402.3003**	302.2115	262.1802	**190.1590**	**230.1903**
heptadeca-8,11-dienoic acid	**388.2846**	288.1958	248.1645	**190.1590**	**230.1903**
hexadeca-7,10-dienoic acid	**374.2690**	274.1802	234.1489	**190.1590**	**230.1903**
pentadeca-6,9-dienoic acid	**360.2533**	260.1645	220.1332	**190.1590**	**230.1903**
tetradeca-5,8-dienoic acid	**346.2377**	246.1489	206.1176	**190.1590**	**230.1903**
heptadeca-8,11-dienal	**372.2897**	272.2009	232.1696	**190.1590**	**230.1903**
hexadeca-7,10-dienal	**358.2741**	258.1852	218.1539	**190.1590**	**230.1903**
pentadeca-6,9-dienal	**344.2584**	244.1696	204.1383	**190.1590**	**2301903**
tetradeca-5,8-dienal	**330.2428**	230.1539	190.1226	**190.1590**	**230.1903**

Table S4 Identified PB oxetane adducts along with their deduced diagnostic fragments (α- and ω-ions) during nanoESI–online–PB–MS/MS–analysis of the reaction mixture of palmitoleic acid (see chapter "Identification"). All detected *m/z* signals are assigned in **bold** and showed a maximum deviation of ± 5 ppm from theoretical values

compound	*m/z* [PB adduct]	*m/z* [α-ion]	*m/z* [ω-ion]
hexadec-9-enoic acid	**376.2846**	262.1802	**204.1747**
pentadec-8-enoic acid	**362.2690**	**248.1645**	**204.1747**
tetradec-7-enoic acid	**348.2533**	234.1489	**204.1747**
tridec-6-enoic acid	**334.2377**	220.1332	**204.1747**
dodec-5-enoic acid	**320.2220**	206.1176	**204.1747**
pentadec-8-enal	**346.2741**	232.1696	**204.1747**
tetradec-7-enal	**332.2584**	218.1539	**204.1747**
tridec-6-enal	**318.2428**	204.1383	**204.1747**
dodec-5-enal	**304.2271**	190.1226	**204.1747**

References

Hammer AK, Emrich NO, Ott J, Birk F, Fraatz MA, Ley JP, Geissler T, Bornscheuer UT, Zorn H (2021) Biotechnological production and sensory evaluation of ω1-unsaturated aldehydes. J. Agric. Food Chem. 69:345–353
